# *Coxiella burnetii* in Wild-caught Filth Flies

**DOI:** 10.3201/eid1406.071691

**Published:** 2008-06

**Authors:** Mark P. Nelder, John E. Lloyd, Amanda D. Loftis, Will K. Reeves

**Affiliations:** * Clemson University, Clemson, South Carolina, USA; † University of Wyoming, Laramie, Wyoming, USA; ‡ Centers for Disease Control and Prevention, Atlanta, Georgia, USA; § US Department of Agriculture, Laramie; 1 Current affiliation: Private practice, Laramie, Wyoming, USA

**Keywords:** Blowfly, Q fever, stable fly, zoo, zoonotic, sheep ked, Coxiella burnetii, Melophagus ovinus, letter

**To the Editor:** *Coxiella burnetii*, the agent of Q fever, is a bacterium and a potential agent of bioterrorism. The most frequent signs of infection in domestic animals are abortion and reduced fertility (*1*). Clinical signs of Q fever in humans vary from mild fevers to pneumonia, hepatitis, or death; atypical cases occur as other disorders, such as cholecystitis (*1*,*2*). Aerosols are the most common route of exposure, but oral transmission occurs (*1*).

Some flies feed on the feces, milk, carcasses, or blood of domestic animals that can be infected with *C*. *burnetii*. These flies regurgitate and defecate when feeding and are mechanical vectors of bacteria (*3*,*4*). Flies have been shown to harbor, mechanically transport, and even support the growth of *C*. *burnetii* (*4*–*6*). It is known that house flies (*Musca domestica*) are possible mechanical vectors of *C*. *burnetii* because this organism survived 32 days in house flies and viable bacteria were shed by flies for 15 days (*4*). There are no studies of *C*. *burnetii* in field-collected flies. To examine the prevalence of *C. burnetii* in field-collected flies, we tested flies from farms, forests, ranches, and zoos.

Flies that develop on animal dung, carcasses, feces, blood, or garbage are often called filth flies. Adult Calliphoridae, Hippoboscidae, Muscidae, and Sarcophagidae were collected from forests, zoos, ranches, and farms (Table). Flies were killed in 95% ethanol or by freezing. DNA was extracted from individual flies as described (*7*,*8*). A distilled water negative control was used for each extraction.

**Table:** Flies from the United States and Dominica assayed for *Coxiella burnetii*, 2004–2007

Species	Collection site and collector	Date of collection	No. positive for *C. burnetii*/no. collected
*Calliphora vicina* (Robineau-Desvoidy)	Elephant dung, Greenville Zoo, Greenville County, SC, USA, by M.P. Nelder	2006 Apr 18	0/1
*Lucilia coeruleiviridis* (Marquart)	Trapped on carrion near Pickens, SC, USA, by K.D. Cobb and W.K. Reeves	2004 Jul 3	1/12
*L. coeruleiviridis* (Marquart)	Elephant dung, Greenville Zoo, Greenville County, SC, USA, by M.P. Nelder	2006 Jul 18	0/13
*L. coeruleiviridis* (Marquart)	Elephant dung, Greenville Zoo, Greenville County SC, USA, by M.P. Nelder	2005 Aug 17	0/3
*L. sericata* (Meigen)	Garbage bin, Greenville Zoo, Greenville County, SC, USA, by M.P. Nelder	2005 Aug 17	3/18
*Melophagus ovinus* (Linnaeus)	Sheep, Bozeman, Gallatin County, MT, USA, by J.E. Lloyd	2007 Jun 27	0/154
*Musca domestica* (Linnaeus)	Cow, Springfield Estate, St. Paul Parish, Dominica, by W.K. Reeves	2005 May 18	0/6
*M. domestica*	Goat pens, petting exibit, Greenville Zoo, Greenville County, SC, USA, by W.K. Reeves and M.P. Nelder	2005 Aug 1	0/5
*M. domestica*	*Colobus* spp. monkey dung; Greenville Zoo, Greenville County, SC, USA, by M.P. Nelder	2005 Oct 19	0/6
*Ravinia stimulans*	Lion dung, Greenville Zoo, Greenville County, SC, USA, by M.P. Nelder	2005 Oct 16	0/10
*Ravinia* new sp.	Lion dung, Greenville Zoo, Greenville County, SC, USA, by M.P. Nelder	2005 Oct 17	0/11
*Stomoxys calcitrans* (Linnaeus)	Goat pens, petting exibit, Greenville Zoo, Greenville County, SC, USA, by W.K. Reeves and M.P. Nelder	2005 Aug 1	0/20
*S. calcitrans*	Fly trap, Greenville Zoo, Greenville County, SC, USA, by M.P. Nelder	2006 Apr 28	0/20
*S. calcitrans*	Cow, Riverbanks Zoo, Richland County, SC, USA, by M.P. Nelder	2006 Apr 5	0/17
*S. calcitrans*	Cow, goats, horses, and llama, Riverbanks Zoo, Richland County, SC, USA, by M.P. Nelder	2006 Apr 6	0/12
*S. calcitrans*	Cattle and elk hay barn, Sybille Canyon, Albany County, WY, USA, by W. Yarnell	2007 July 14–Aug 3	1/55

Individual DNA samples were tested, in duplicate, with a previously described TaqMan assay with a lower limit of detection of 1 *C*. *burnetii* organism (*8*). Positive and negative controls were used for all assays. Positive flies were verified by PCR and sequencing of 16S rRNA gene as described (*9*). Vouchers for each insect species were deposited in the Clemson University Arthropod Collection (Clemson, SC, USA), the University of Georgia Museum of Natural History (Athens, GA, USA), or the University of Wyoming Insect Collection (Laramie, WY, USA).

Five of 363 flies were positive for *C*. *burnetii* DNA (Table). These flies included *Stomoxys calcitrans*, in which the adults feed on animal and human blood, and the blowflies *Lucilia coeruleiviridis* and *L*. *sericata*. *C*. *burnetii*–positive flies were obtained from carrion (1/12, 8.3%), a garbage bin of elephant feces (3/18, 16.7%), and a barn at a ranch (1/55, 1.8%). We sequenced 1,100 bp of the 16S rRNA gene from select DNA extracts, which were 99% identical with that of *C*. *burnetii* strain NC 002971.

We detected DNA from *C*. *burnetii* in flies from a zoo, a ranch, and carrion in a forest. Laboratory data on house flies, which shed live *C*. *burnetii* for 15 days after exposure, suggest that related flies (e.g., *S*. *calcitrans* and *Lucilia* spp.) might also harbor viable *C*. *burnetii*. On the basis of our field data, *S*. *calcitrans* and *Lucilia* spp. should be studied as mechanical vectors of *C*. *burnetii*. Unlike many enteric bacteria, which require large inocula to cause disease, *C*. *burnetii* can be infectious at the level of 1 bacterium (*10*). If flies transmit *C*. *burnetii,* they pose an additional threat to human and animal health.

The role of the sheep ked (*Melophagus ovinus*) in maintenance or transmission of *C*. *burnetii* is unknown. This fly is an obligate ectoparasite of sheep. It feeds on sheep blood, and feces from sheep keds can accumulate in the wool of sheep. Testing of sheep keds from infected sheep would help understand whether keds play a role in the epidemiology of *C*. *burnetii*.
